# Trapped in time

**DOI:** 10.7554/eLife.90008

**Published:** 2023-07-14

**Authors:** Kenneth De Baets, Karina Vanadzina, James Schiffbauer

**Affiliations:** 1 Institute of Evolutionary Biology, Faculty of Biology, University of Warsaw Warsaw Poland; 2 Department of Geological Sciences, University of Missouri Columbia United States

**Keywords:** fossil, amber, nematode, insect, parasitism, Cretaceous, ecological interactions, paleoparasitology, None

## Abstract

Analysis of specimens preserved in amber from the Cretaceous period suggests that nematodes changed their host preference towards insects with a complete metamorphosis more recently.

**Related research article** Luo C, Poinar GO, Xu C, Zhuo D, Jarzembowski EA, Wang B. 2023. Widespread mermithid nematode parasitism of Cretaceous insects. *eLife* **12**:e86283. doi: 10.7554/eLife.86283.

Fossils trapped in amber can reveal astonishing insights about the lives of ancient organisms. Amber can preserve life forms in incredible detail, from their three-dimensional anatomy down to cellular level. It also often catches animals in the middle of an action, like copulation, feeding or pollination, thus providing valuable information about the behavior of a species.

Fossils may also reveal insights into the relationship between parasites and their hosts and how they coevolved. Parasitic relationships, where one species benefits from another – and usually to the other’s detriment – are widespread and crucial for ecosystems. A better understanding of how these relationships have changed over time is thus vital for predicting future changes to diversity (Farrell et al., 2021).

Scientists often rely on phylogenetic analysis and host-distribution data to study these interactions. However, the potential of fossils has so far been overlooked when studying the association between parasites and their hosts (De Baets et al., 2021). Now, in eLife, Bo Wang and colleagues from various research institutes in China and the United States – including Cihang Luo as first author – report how they used fossil records to study the coevolution of nematodes and their invertebrate (Luo et al., 2023).

Nematodes, also known as roundworms, can be found in most habitats across the globe and multiple lineages have independently acquired a parasitic lifestyle. Some parasitic roundworms using plants as hosts have existed at least since the Devonian period around 408 million years ago (Figure 1). The oldest lineages exploiting animals can be found as eggs in Triassic vertebrate coprolites, which may indicate that lineages exploiting invertebrates might have evolved by that time. However, fossils of intact soft-bodied worms are rare, as they usually decay before fossilization sets in. Luo et al. therefore decided to focus their attention on the Mermithidae family, a group of nematodes that eventually kill their insect hosts once they emerge, making it easy to study their life histories.

**Figure 1.**
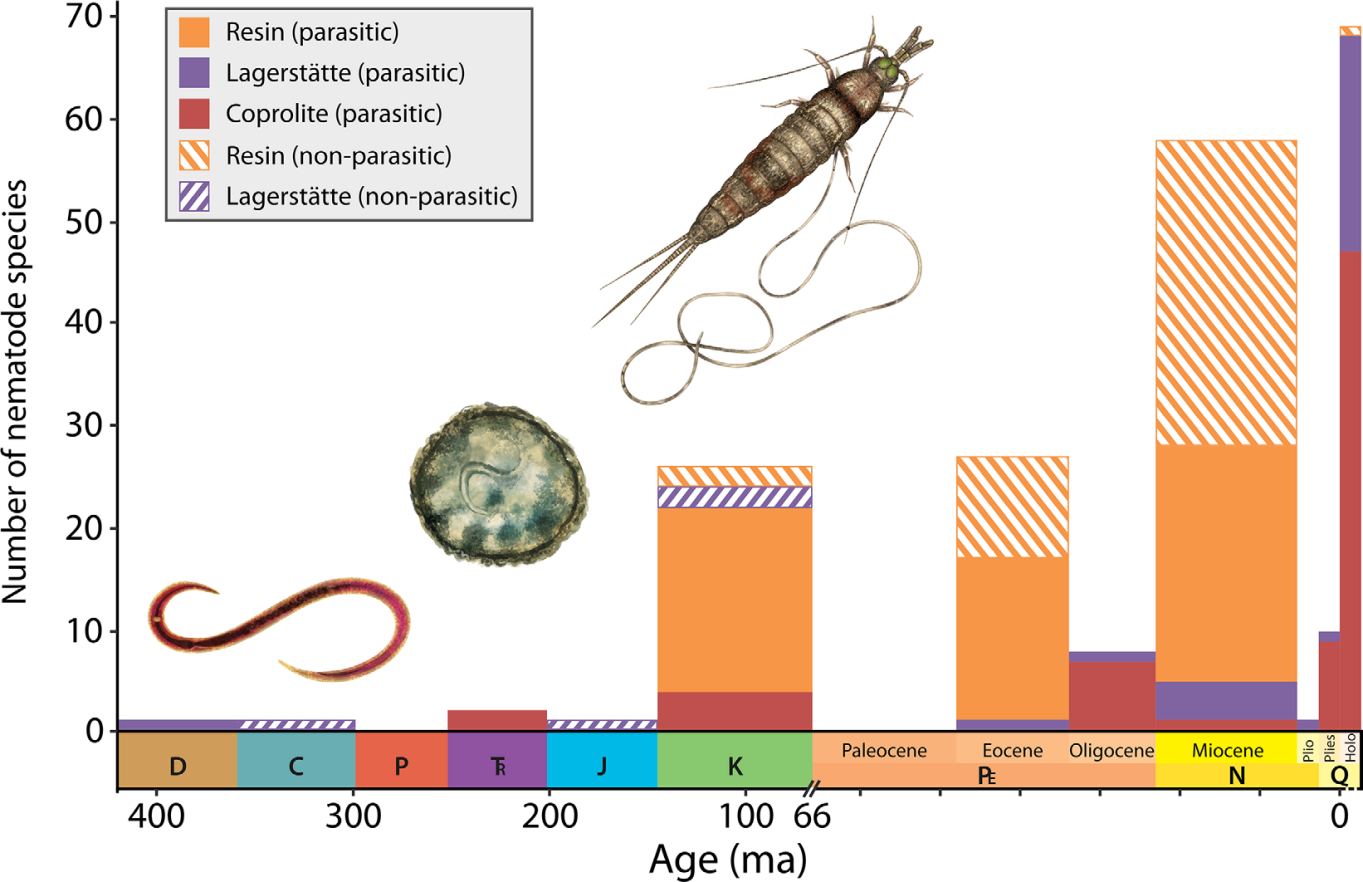
Number of reported parasitic and nonparasitic nematode fossil species from the Devonian through to Holocene. Nematodes are roundworms that can form parasitic relationships (solid bars) with their hosts. One of the oldest known examples – nematodes that parasitize plants – dates back to the Devonian period, around 408 million years ago (ma). The figure shows reconstructions (not to scale) of the fossil nematodes *Palaeonema phyticum* (left) from early land plants found in well preserved fossil beds known as Lagerstätte (purple bars); *Ascarites priscus* (middle) preserved in Cretaceous vertebrate coprolite ca. 125 ma (red bars); and *Cretacimermis incredibilis* (right) exiting a bristletail preserved in Cretaceous amber described by Luo et al., 2023 (orange bars). Data updated from De Baets et al., 2021. Abbreviations: D: Devonian, C: Carboniferous, P: Permian, T: Triassic, J: Jurassic, K: Cretaceous, PE: Paleocene, N: Neogene, Q: Quaternary, Plio: Pliocene, Pleis: Pleistocene, Holo: Holocene.

Illustrations provided by Franz Anthony (CC BY 4.0).

By analyzing several fossil samples, the team could augment the number of described mermithid species from Cretaceous amber to 13, identifying nine species of mermithids with an age around 100 million years which had not been previously recorded. This doubles the currently known diversity of parasitic nematodes alive during the Cretaceous from nine to 18 species and suggests that parasitism by mermithids was rather widespread during this time, which probably helped regulate insect populations.

Luo et al. further provided the first fossil records of seven host associations, including three arthropod groups that are not recognized hosts today: bristletails, bark lice and extinct planthoppers. Additional amber samples further revealed four modern mermithid-host associations not yet known from the fossil record including dragonflies, earwigs, crickets and cockroaches.

To further explore the evolutionary relationship between nematodes and their hosts, Luo et al. looked at data records from three well-known amber periods: the mid-Cretaceous, the Eocene and the Miocene. This revealed a major shift in host preference over time. Nematodes in the Cretaceous period preferred insects that lacked complete metamorphosis, while roundworms from the Eocene and Miocene mainly used insects with a complete metamorphosis (80%).

Similar to many other parasitic worms, mermithids prefer aquatic or moist environments but have evolved a strategy to infect land-living arthropods (which would also frequent water sources) as juveniles; the nematodes then kill their hosts when approaching adulthood, so they can continue developing and reproducing in aquatic or humid soil environments (Ni et al., 2021). Due to this parasitoid strategy, mermithids have a tight relationship with their host, but their initial host preferences remain poorly known.

The study of Luo et al. adds to previous research indicating important changes in the diversity of strategies of parasitoids exploiting insects from the Cretaceous period to present day (Labandeira and Li, 2021). It is tempting to attribute this shift to a change in the diversity of host species, which saw some major arthropod lineages becoming extinct and ones appearing during the Cretaceous and the Paleogene period. However, several other factors could also contribute to host preferences, including the variety of sampled paleoenvironments or differences in the quality of preservation or collection practices (Penney, 2016; Solórzano Kraemer et al., 2018).

In the past, researchers have mainly studied amber fossils with the aim of discovering new insect species, leaving nematodes – including mermithids – and other potential hosts undersampled or understudied (Schmidt et al., 2010; Stilwell et al., 2020; Košulič and Mašová, 2019). Further investigations will require systematic sampling and global collaboration among all relevant communities and research institutions, along with sufficient funding. Moreover, since nematodes are less likely to remain fully accessible in amber, it is challenging to study morphological traits that could identify different species. Recent advancements in microscopy and tomography techniques offer promising opportunities to overcome this challenge and to visualize the full morphology of nematodes (Penney, 2016).

Mermithid nematodes, with their distinct physique and the characteristic damage they cause when exiting their hosts, serve as an intriguing model to study evolutionary host-parasite dynamics. Luo et al. show the potential of studying amber deposits to document ancient shifts in host preferences, from the Mesozoic to the present, and demonstrate the advantages of investigating ancient host-parasite relationships to better understand evolutionary patterns and estimate the extinction risk of modern species (Ni et al., 2021; Mulvey et al., 2022).
